# Standardisation of disk diffusion results for antibiotic susceptibility testing using the sirscan automated zone reader

**DOI:** 10.1186/1471-2180-13-225

**Published:** 2013-10-08

**Authors:** Michael Hombach, Reinhard Zbinden, Erik C Böttger

**Affiliations:** 1 Institut für Medizinische Mikrobiologie, Universität Zürich, Gloriastrasse 30/32, 8006 Zürich, Switzerland

**Keywords:** Inhibition zone diameter, Kirby-Bauer, Automation

## Abstract

**Background:**

Standardisation of disk diffusion readings could improve reproducibility and accuracy of antibiotic susceptibility testing (AST). This study evaluated accuracy, reproducibility, and precision of automated inhibition zone reading using the “Sirscan automatic” zone reader (i2a, Perols Cedex, France).

**Results:**

In a first step we compared Sirscan results with manual calliper measurements for comparability and accuracy. Sirscan readings were checked and adjusted on-screen as recommended by the manufacturer. One hundred clinical bacterial isolates representing a broad spectrum of organisms routinely isolated in a clinical laboratory were tested, and zone diameter values and interpretation according to EUCAST guidelines were compared. In a second step we analysed, whether fully automated zone reading can decrease standard deviation of diameter measurements and, thus, improve reproducibility and precision of the disk diffusion method. Standard deviations of manual measurements, on-screen adjusted Sirscan measurements, and fully automated Sirscan readings were compared for 19 repeat independent measurements of inhibition zones of *S. aureus* ATCC 29213, *E. coli* ATCC 25922, and *P. aeruginosa* ATCC 27853 (EUCAST quality control strains).

On-screen adjusted Sirscan and calliper measurements displayed high comparability. No significant differences were detected comparing the results of both reading methods. Standard deviations of inhibition zone diameters were significantly lower for fully automated Sirscan measurements compared with both adjusted Sirscan readings and the manual method, resulting in better reproducibility and precision of the automated readings.

**Conclusions:**

Our results indicate that fully automated zone reading can further improve standardisation of AST by decreasing standard deviation and, thus, improve precision of inhibition zone diameter results.

## Background

Disk diffusion has been the mainstay for antimicrobial susceptibility testing (AST) in most clinical microbiological laboratories since Bauer, Kirby et al. first described this technique in the 1960s [1]. During the past decade automated AST microdilution systems based on determination or extrapolation of minimal inhibitory concentrations have been introduced in the diagnostic market, e.g. systems like the Vitek 2 (BioMérieux), Phoenix (Becton-Dickinson), or Microscan (Siemens Healthcare Diagnostics).

The main advantages of commercial microdilution systems including automated reading and rapidity are compromised by the still lower sensitivities in the detection of important resistance mechanisms compared with the disk diffusion method, e.g. inducible macrolide-lincosamide-streptogramin resistance (MLS_B_-Type), extended spectrum beta-lactamases (ESBL), and AmpC beta-lactamases [2-5]. In addition, some combinations of resistance mechanisms are not reliably detected by automated microdilution systems e.g. ESBL in *Enterobacteriaceae* isolates co-producing chromosomally- or plasmid-encoded AmpC beta-lactamases [6]. The sensitivity for detection of resistance mechanisms largely depends on the composition of the antibiotic drug panel used in the automated microdilution systems, which cannot be changed or modified by the user [2,7].

The disk diffusion method readily permits detection of inducible phenotypes and most combinations of resistance mechanisms including ESBL and AmpC co-production. The antibiotic panel composition is flexible and enables the clinical laboratory to readily adjust the composition of panels to its needs [8,9]. Disadvantages of the disk diffusion method are its labour cost due to manual measurements and manual data documentation, and the investigator dependence and variation of results [10].

During the past decade several systems have been developed to automate disk diffusion readings. Systems like Sirscan (i2a, Montpellier, France), OSIRIS and ADAGIO (both BIO-RAD, Marne La Coquotte, France), Oxoid Aura (Oxoid Ltd., Basingstoke, UK), or BIOMIC (Giles Scientific Inc., Santa Barbara, California, USA) are able to automatically read inhibition zone diameters and incorporate expert systems for AST interpretation. These systems allow fully automated (Sirscan) or semi-automated reading (ADAGIO, Aura, BIOMIC), documentation and data interpretation using expert systems. The few studies available investigating the performance of automated zone reading systems indicate a high agreement with standard manual calliper (correlation coefficients ranging from 0.91 to 0.96) resulting in only few susceptibility categorisation errors [10-15]. However, some systems are no longer available (OSIRIS, Oxoid Aura), or have reported practical problems for routine use (BIOMIC) [16].

No studies are available investigating, if and to which extent fully automated zone reading is able to facilitate standardisation of inhibition zone diameter measurements. High reproducibility and low variation of results become even more important in the light of the new CLSI and EUCAST AST guidelines that contain smaller intermediate susceptibility categories or, in case of EUCAST, have even partially abandoned the use of the intermediate category. Directly adjacent susceptible and resistant categories lead to a higher frequency of major and very major errors (i.e. susceptible to resistant, resistant to susceptible) simply due to technical reasons, i.e. variation of individual measurements [17-19].

This study aimed at comparing the fully automated Sirscan with standard calliper measurements assessing: i) The agreement of inhibition zone diameter results (comparability), ii) The frequency of discrepancies in susceptibility categorisation (accuracy), and iii) Variation of repeat diameter measurements (reproducibility and precision).

## Methods

### Clinical isolates

One hundred clinical bacterial isolates were selected as a representative sample of organisms routinely isolated in the clinical microbiological laboratory. Bacterial strains comprised 21 *Escherichia coli*, 17 *Staphylococcus aureus*, 17 *Enterococcus* spp., 16 coagulase-negative staphylococci, 9 *Pseudomonas aeruginosa*, 7 *Klebsiella pneumoniae*, 4 *Enterobacter* spp., 2 *Serratia* spp., 2 *Stenotrophomonas maltophilia*, 2 *Acinetobacter* spp., 2 *Proteus* spp., and 1 *Citrobacter* spp. For reproducibility testing, *Staphylococcus aureus* ATCC 29213, *Escherichia coli* ATCC 25922, and *Pseudomonas aeruginosa* ATCC 27853 (EUCAST quality control strains) were used. The following non-duplicate clinical isolates with confirmed resistance mechanisms were included to test for adequate detection of individual resistance mechanisms by the Sirscan instrument: 117 Extended-spectrum beta-lactamase (ESBL) producing *Enterobacteriaceae* isolates (105 CTX-M type, 10 SHV-ESBL-type, and 2 TEM-ESBL type), 38 AmpC producing *Enterobacteriaceae* isolates (24 plasmid-encoded CIT-type AmpC, 2 plasmid-encoded DHA-type AmpC, and 12 *E. coli* isolates harboring *ampC* promoter mutations leading to overexpression of AmpC), 13 carbapenemase producing *Enterobacteriaceae* isolates (6 KPC type, 3 VIM type, 2 OXA-48 type, 1 NDM-1 type, 1 GIM-1 type), 17 vancomycin-resistant enterococci (VRE) isolates, and 50 methicillin-resistent *S. aureus* (MRSA) isolates [5,9].

### Susceptibility testing

Disk diffusion testing was done according to the 2011 guidelines of the European Committee of Antimicrobial Susceptibility Testing (EUCAST) using standard antibiotic disks (i2a, Perols Cedex, France) and Mueller-Hinton agar plates (BD, Franklin Lakes, NJ). All measurements except those for investigator dependence were done by the same experienced laboratory technician to eliminate *inter*-person bias. In parallel, the disk diffusion Mueller-Hinton agar plates were measured with the Sirscan instrument (i2a, Perols Cedex, France) and manually using a standard calliper. Sirscan measurements were checked and corrected on-screen by the laboratory technician as recommended by the manufacturer. Standard deviations of zone diameter measurements were calculated from 19 independent and blinded readings by 19 experienced persons using antibiotic disk diffusion inhibition zones of *S. aureus* ATCC 29213, *E. coli* ATCC 25922, and *P. aeruginosa* ATCC 27853 (EUCAST quality control strains). Discrepancies of manual and Sirscan readings were categorised as follows: Discrepancies resulting in erratic assignment of bacterial isolates to adjacent interpretative categories (susceptible to intermediate, intermediate to susceptible, intermediate to resistant, resistant to intermediate) were referred to as “minor discrepancies”. Erroneous categorisation of true-susceptible isolates as resistant (considering the manual method as the gold standard) were referred to as “major discrepancies”. Categorisation of true-resistant isolates as susceptible (considering the manual method as the gold standard) were referred to as “very major discrepancies”.

The following parameters were used to test for the presence of individual resistance mechanisms using Sirscan readings: ESBL-screening was done using EUCAST clinical breakpoints for non-susceptibility to cefpodoxime, and/or ceftazidime, and/or cefotaxime, ceftriaxone, and/or cefepime, AmpC and MRSA-screening was done using EUCAST clinical breakpoints for non-susceptibility to cefoxitin, carbapenemase-screening was done using EUCAST clinical breakpoints for non-susceptibility to ertapenem, and/or meropenem, and/or imipenem, and VRE-screening was done using EUCAST clinical breakpoints for non-susceptibility to vancomycin [18].

All inhibition zone diameter results were recorded by the Sirweb software (i2a, Perols Cedex, France) and statistical parameters were calculated with the Microsoft Excel 2010 Software (Microsoft Corp., Redmond, WA).

### Antibiotic drugs

Different antibiotic drug panels were tested for Gram-negative rods, *Staphylococcus* spp., and *Enterococcus* spp. Antibiotic drugs tested for Gram-negative rods comprised ampicillin, amoxicillin/clavulanic acid, piperacillin/tazobactam, cefuroxime, cefpodoxime, ceftriaxone, ceftazidime, cefotaxime, cefepime, cefoxitin, ertapenem, imipenem, meropenem, amikacin, gentamicin, tobramycin, nalidixic acid, ciprofloxacin, levofloxacin, nitrofurantoin, and trimethoprim-sulfamethoxazole. Antibiotic drugs tested for *Staphylococcus* spp. comprised penicillin, cefoxitin, amikacin, gentamicin, tobramycin, ciprofloxacin, levofloxacin, rifampicin, erythromycin, clindamycin, and trimethoprim-sulfamethoxazole. Antibiotic drugs tested for *Enterococcus* spp. comprised ampicillin and vancomycin.

## Results

Mean differences of inhibition zone diameter measurements were less than 2 mm for all antibiotic classes and bacterial groups comparing on-screen adjusted Sirscan readings (manufacturer recommended) and manual readings for the 100 clinical strains (Table 1), with the exception of ampicillin and *Enterococcus* spp. that showed a mean difference of 2.5 mm. On average, mean differences of all antibiotic drug classes were higher for *Staphylococcus* spp. and *Enterococcus* spp. than for Gram-negative rods (1.2 mm, 1.7 mm, and 0.9 mm, respectively, see Table 1). For Gram-negative rods the carbapenems showed mean differences of inhibition zone diameters above average, for staphylococci clindamycin, penicillins, and quinolones showed mean differences of inhibition zone diameters higher than the average (Table 1).

**Table 1:** Mean differences of zone diameters measurements as determined by calliper and Sirscan on-screen adjusted

**Drug or drug class**		**Zone diameter mean difference (mm)**	
	**Gram-negative rods**	***Staphylococcus *spp.**	***Enterococcus *spp.**
Penicillins	0.9	1.4	2.5
Cephalosporins	1		
Carbapenems	1.4		
Aminoglycosides	0.6	1.3	
Quinolones	0.9	1.4	
Trimethoprim-Sulfamethoxazole	0.8	0.9	
Rifampicin		1.1	
Glycopeptides			0.8
Cefoxitin		0.7	
Clindamycin		1.6	
All antibiotics	0.9	1.2	1.7

Antibiotic drug classes / drugs tested for Gram-negative rods comprised ampicillin, amoxicillin/clavulanic acid, piperacillin/tazobactam (Penicillins), cefuroxime, cefpodoxime, ceftriaxone, ceftazidime, cefotaxime, cefepime, cefoxitin, (cephalosporins), ertapenem, imipenem, meropenem (carbapenems), amikacin, gentamicin, tobramycin (aminoglycosides), nalidixic acid, ciprofloxacin, levofloxacin (quinolones), and trimethoprim-sulfamethoxazole. Antibiotic drug classes / drugs tested for *Staphylococcus* spp. comprised penicillin (penicillins), cefoxitin, amikacin, gentamicin, tobramycin (aminoglycosides), ciprofloxacin, levofloxacin (quinolones), rifampicin, erythromycin, clindamycin, and trimethoprim-sulfamethoxazole. Antibiotic drugs tested for *Enterococcus* spp. comprised ampicillin (penicillins) and vancomycin (glycopeptides).

The relative deviations of inhibition zone diameter measurements (higher or lower inhibition zone diameter values of one method compared to the other) were almost equally distributed between on-screen adjusted Sirscan and manual measurements (Table 2). *Enterococcus* spp. constituted an exception as lower zone diameters with the Sirscan were observed in 53% of the cases. However, no major or very major discrepancies resulted from these deviations comparing on-screen adjusted Sirscan with manual calliper measurements that were considered as the gold standard (using EUCAST 2011 AST guidelines) [18]. Reported AST results with the on-screen adjusted Sirscan system were as accurate as the currently recommended manual method.

**Table 2:** Relative deviation of zone diameter values and resulting discrepancies of the Sirscan (on-screen adjusted) and manual calliper measurements

	**Relative deviation of zone diameters values**			**Discrepancies**		
	**(% of all measurements)**			**(% of all Sirscan measurements)**		
	**Sirscan < calliper**	**Sirscan = calliper**	**Sirscan > calliper**	**minor**	**major**	**very major**
Gram-negative rods	19	45	36	1.27	0	0
*Staphylococcus* spp.	27	37	36	0.94	0	0
*Enterococcus* spp.	53	35	12	0	0	0

For discrepancy analysis manual calliper measurements were regarded as the gold standard. Sirscan values were on-screen adjusted by an experienced person as recommended by the manufacturer.

All isolates with confirmed resistance mechanisms, i.e. ESBL-, AmpC, and carbapenemase producing *Enterobacteriaceae* isolates, VRE, and MRSA were adequately detected using Sirscan readings with two exceptions: One CIT-type AmpC producing isolate, and one MRSA isolate showing cefoxitin inhibition zone diameters of 21 mm (corresponding non-susceptible EUCAST breakpoint <19 mm), and 22 mm (corresponding non-susceptible EUCAST breakpoint <22 mm), respectively. Inhibition zone diameters could subsequently be confirmed by manual reading.

The reproducibility and precision of repeat readings by 19 experienced persons were significantly higher with fully automated Sirscan readings compared with the manufacturer recommended on-screen adjusted Sirscan readings and manual calliper measurements (Table 3). The average standard deviations for *S. aureus* ATCC 29213, *E. coli* ATCC 25922, and *P. aeruginosa* ATCC 27853 were 0.8 mm, 0.7 mm, and 0.6 mm (Sirscan fully automated), and 1.6 mm, 1.4 mm, and 0.8 mm (manual readings). Standard deviations of on-screen adjusted Sirscan readings were comparable to the manual method (1.3 mm, 1.4 mm, and 1.0 mm, for *S. aureus* ATCC 29213, *E. coli* ATCC 25922, and *P. aeruginosa* ATCC 27853, respectively). The lower standard deviation of fully automated Sirscan readings was pronounced for certain antibiotics (Table 3): E.g. for trimethoprim-sulfamethoxazole and *S. aureus* ATCC 29213 (0.9 mm versus 4.7 mm for fully automated Sirscan and manual readings, respectively) or for trimethoprim-sulfamethoxazole and nitrofurantoin in *E. coli* ATCC 25922 (0.4 and 0.5 mm versus 0.9 and 1.6 mm for fully automated Sirscan and manual readings, respectively).

**Table 3:** Comparison of standard deviations of measurements with calliper, the Sirscan system adaped on-screen by the human eye and the Sirscan fully automated mode

***S. aureus *ATCC 29213**																					
	**TOB**	**AK**	**CN**	**CIP**	**LEV**	**P**	**FOX**	**E**	**DA**	**SXT**	**RA**	**average**									
**EUCAST QC range**	20-26	18-24	19-25	21-27	23-29	12-18	24-30	23-29	23-29	29-32	30-36										
**Sirscan fully automated**																					
**Mean value**	23.2	23.4	23.6	27.8	28.1	15.9	25.0	27.8	29.4	29.4	32.7	**26.0**									
**Standard deviation**	0.8	0.5	0.8	0.9	1.3	0.3	1.2	0.8	0.7	0.9	1.0	0.8*									
**Sirscan on-screen adjusted**																					
**Mean value**	24.7	25.5	25.2	27.8	29.5	15.8	26.1	30.4	29.9	29.8	33.6	**27.1**									
**Standard deviation**	1.2	0.7	1.4	1.7	1	0.4	0.9	2.2	3	1.3	0.7	**1.3**									
**Calliper**																					
**Mean value**	23.4	23.2	23.8	22.7	27.2	17.2	26.1	26.2	26.7	26.2	32.9	**25.1**									
**Standard deviation**	1	1.6	1.2	1.1	2	0.6	0.8	1.1	1.2	4.7	2.1	1.6*									
***E. coli *ATCC 25922**																					
	**TOB**	**AK**	**CN**	**NA**	**NOR**	**CIP**	**LEV**	**AM**	**AMC**	**TPZ**	**CXM**	**CAZ**	**CTX**	**CPD**	**CRO**	**FEP**	**MEM**	**ETP**	**SXT**	**NF**	**average**
**EUCAST QC range**	18-26	19-26	19-26	22-28	28-35	30-40	29-37	16-22	18-24	21-27	20-26	23-29	25-31	23-28	29-35	31-37	28-34	29-36	23-29	17-23	
**Sirscan fully automated**																					
**Mean value**	22.4	20.6	20.4	25.9	28.1	28.4	28.4	20.6	21.8	23.3	24.8	26.0	27.5	24.7	29.3	31.0	29.2	34.0	26.3	17.5	**25.5**
**Standard deviation**	1.1	0.8	0.5	0.8	0.6	1.0	0.7	0.9	0.6	0.4	0.4	0.0	0.5	0.5	0.7	0.7	0.9	0.8	0.4	0.5	0.7*
**Sirscan on-screen adjusted**																					
**Mean value**	23.2	24.5	25.1	25.9	34	37.3	35.4	25.9	23.3	27.6	26.1	27.8	31.1	29.3	32.3	35	35.9	34.3	28.4	24.8	**29.4**
**Standard deviation**	1.5	1.3	1.4	1.3	2.4	1.6	1.5	1.8	1	1	1.2	1.2	1.9	1.2	1.3	1	1.2	1.2	1.4	1	**1.4**
**Calliper**																					
**Mean value**	22.4	24.4	23.3	27.6	31.1	34.1	31.5	22.3	25.1	25.7	24.8	25.5	28.5	27.6	30.2	33.7	23.9	34.3	27.3	17.4	**27.0**
**Standard deviation**	1.1	2.5	1.2	1.3	1	1.6	1.5	1.2	0.7	1.1	1.2	1	1.5	2.4	1.4	1.4	1.3	2.4	0.9	1.6	1.4*
***P. aeruginosa *ATCC 27853**																					
	**TOB**	**AK**	**CN**	**LEV**	**FEP**	**CAZ**	**TPZ**	**IPM**	**MEM**	**average**											
**EUCAST QC range**	19-25	18-26	16-21	19-26	24-30	21-27	23-29	20-28	27-33												
**Sirscan fully automated**																					
**Mean value**	24	24.9	21.5	28	27.8	22.9	25.3	23.6	29.9	**25.3**											
**Standard deviation**	0.8	0.7	1.4	0.6	0.4	0.3	0.7	0.5	0.3	**0.6**											
**Sirscan on-screen adjusted**																					
**Mean value**	23.2	25.2	22	27.8	26.6	22.2	24.5	25	26.5	**24.8**											
**Standard deviation**	0.8	1	0.9	1.3	1.4	0.9	1.2	0.5	0.6	**1.0**											
**Calliper**																					
**Mean value**	23.5	25.0	21.6	25.9	25.8	22.2	23.9	24.9	26.4	**24.4**											
**Standard deviation**	0.6	0.7	0.5	1.2	0.9	0.8	1.1	0.6	0.9	**0.8**											

Standard deviations of repeat measurements of *S. aureus* ATCC 29213 and *E. coli* ATCC 25922 were significantly lower with fully automated Sirscan readings as compared to manual calliper measurements indicating better reproducibility and precision of Sirscan readings. Asterisks indicate statistically significant differences (p<0.05) of mean standard deviations using the paired t-test. Measurements were done independently and double-blinded by 19 experienced persons (technicians and laboratory physicians) with the same disk diffusion plates of EUCAST quality control strains of *S. aureus* ATCC 29213, *E. coli* ATCC 25922, and *P. aeruginosa* ATCC 27853. Measurements of the Sirscan fully automated mode comprise 19 independent measurements of the panels. QC, quality control; AM, ampicillin; AMC, amoxicillin-clavulanic acid; AK, amikacin; CAZ, ceftazidime; CIP, ciprofloxacin; CN, gentamicin; CPD, cefpodoxime; CRO, ceftriaxone; CTX, cefotaxime; CXM, cefuroxime; DA, clindamycin; E, erythromycin; ETP, ertapenem; FEP, cepefim; FOX, cefoxitin; IPM, imipenem; LEV, levofloxacin; MEM, meropenem; NA, nalidixic acid; NF, nitrofuratoine; NOR, norfloxacin; P, penicillin G; RA, rifampicin; SXT, trimethoprim-sulfamethoxazole.

Examples of measurement variations are shown in Table 4 as scattergram illustrations: 6 / 19 manual calliper measurements for nitrofurantoin in *E. coli* ATCC 25922 were lower than the EUCAST recommended quality control range. Adjusted Sirscan readings showed slightly lower variation, but 6 / 19 nitrofurantoin measurements were still out of the quality control range. Sirscan measurements for nitrofurantoin in the fully automated mode showed significantly lower variation and all were in the quality control range. A comparable pattern was seen with ertapenem for *E. coli* ATCC 25922 and amikacin for *S. aureus* ATCC 29213. The most prominent effect of fully automated readings on standard deviation of zone diameter measurements was observed for trimethoprim-sulfamethoxazole and *S. aureus* ATCC 29213: 14 / 19 calliper measurements were not in the EUCAST quality control range and inter-person variation was high (lowest value 18 mm, highest value 34 mm, i.e. a range of 18 mm). In contrast, fully automated Sirscan readings had a range of 4 mm and only 4 out of 19 values were 1 mm out of the quality control range.

**Table 4:** Examples of scattergrams of inhibition zone measurements with calliper, the Sirscan system adaped on-screen by the human eye and the Sirscan fully automated mode


**Nitrofurantoin, *E. coli *ATCC 25922**																				
Diameter (mm)	**15**	**16**	***17***	***18***	***19***	***20***	***21***	***22***	***23***	**24**										
Sirscan fully automated			9	10																
Sirscan on-screen adjusted		6	4	5	2	2														
Calliper	3	3	4	3	5	1														
**Ertapenem, *E. coli *ATCC 25922**																				
Diameter (mm)	**28**	***29***	***30***	***31***	***32***	***33***	***34***	***35***	***36***	**37**	**38**									
Sirscan fully automated						3	7	9												
Sirscan on-screen adjusted					1		4	6	2	3	3									
Calliper	1			1	1	4	3	1	5	3										
																				
**Trimethoprim-Sulfamethoxazole, *S. aureus *ATCC 29213**																				
Diameter (mm)	**18**	**19**	**20**	**21**	**22**	**23**	**24**	**25**	**26**	**27**	**28**	***29***	***30***	***31***	***32***	**33**	**34**	**35**	**36**	**37**
Sirscan fully automated											4	6	7	2						
Sirscan on-screen adjusted											1	4	3	7	4					
Calliper	2	1	1			1		1	2	3	2	1	2	1	1		1			
**Amikacin, *S. aureus *ATCC 29213**																				
Diameter (mm)	**17**	***18***	***19***	***20***	***21***	***22***	***23***	***24***	**25**											
Sirscan fully automated						7	12													
Sirscan on-screen adjusted						1	6	8	4											
Calliper		1				5	3	7	3											

Measurements were done independently and double-blinded by 19 experienced persons (technicians and laboratory physicians) with the same disk diffusion plates of EUCAST quality control strains of S. aureus ATCC 29213, and E. coli ATCC 25922. Measurements of the Sirscan fully automated mode comprise 19 independent measurements of the panels. EUCAST quality control ranges are indicated in italics.

## Discussion

Automation of inhibition zone readings was developed to avoid disadvantages of disk diffusion AST such as high manual workload, laborious data documentation, and low speed of manual readings. Our results show excellent comparability of on-screen adjusted automated measurements using the Sirscan instrument compared with the manual calliper method for a broad range of species representing the most common isolates in a routine clinical microbiological laboratory (Table 1). The present results are in agreement with other studies that found a high correlation of Sirscan and manual measurements [12,13]. Relative deviations of Sirscan and manual measurements were almost equally distributed pointing to random deviations rather than systematical errors (Table 2). Neither method tended to systematically higher or lower diameter measurements compared with the other with the exception of *Enterococcus spp*., which tended to produce lower zone diameters with the Sirscan (53% of the cases, Table 2) - most probably because the faint bacterial growth was better visible on the computer screen than by the unaided eye. However, this did not result in interpretation discrepancies (Table 2).

Most important, on-screen adjusted automation of disk diffusion readings did not result in an increased frequency of susceptibility categorisation errors. The results of this study showed no major and very major discrepancies occurring with on-screen adjusted Sirscan readings when compared to manual measurements serving as the gold standard. Other authors found low numbers of major and very major errors with the Sirscan system as well [12,13]. Isolates with confirmed resistance mechanisms such as ESBL, AmpC, carbapenemases, VRE, or MRSA were reliably detected except for two isolates showing inhibition zone diameters close to the EUCAST breakpoint. However, both isolates would have been missed by manual reading, too.

Reproducibility and precision of diameter measurements are critical for AST interpretation and antimicrobial therapy. Previous investigations have focused on the correlation of manual and automated measurements using systems like Sirscan, OSIRIS, BIOMIC, or Oxoid Aura [12-16,20]. While correlation of manual and automated systems is well established, we here used a fully automated system to assess, if automated reading is principally able to decrease standard deviation of measurements and, thus, can increase precision. This is of particular importance given the changes in recent EUCAST and, in part, CLSI AST guidelines to decrease or even abandon the intermediate AST zone [19].

Investigator dependence of manual measurements with the disk diffusion method is partly due to non-standardised conditions such as ambient light, angle of vision, reading plates from top or bottom, or physical and mental condition of the investigator. The Sirscan analysis software reads under standardised light, positioning and background conditions. The lack or downsizing of the intermediate category by CLSI and/or EUCAST 2011/12 guidelines enhances the probability of major and very major errors of repeat measurements since susceptible and resistant categories lie directly adjacent to each other [17-19]. Standardisation of measurements with concomitant lower standard deviations will facilitate consistent AST reports for repeatedly tested strains, or for ASTs of one strain isolated from multiple patient samples. The reproducibility of fully automated Sirscan readings without human interaction (on-screen adjustments) was significantly higher compared with manual calliper measurements. The average standard deviation for repeat measurements of *E. coli* ATCC 25922 and *S. aureus* ATCC 29213 inhibition zones was reduced by half using the fully automated reading mode. If, however, Sirscan readings were adjusted on-screen, standard deviations were not significantly lower (Table 3). For *P. aeruginosa* ATCC 27853 the reduction of standard deviations was less pronounced, probably because calliper measurements already showed a low standard deviation of 0.8 mm. This may originate from the pyoverdin-pigmented growth of *P. aeruginosa* ATCC 27853 that allows a more precise measurement of zone edges by the unaided human eye. In contrast, compounds forming fuzzy zone edges showed high standard deviations with manual readings, e.g. trimethoprim-sulfamethoxazole, ertapenem, or cefpodoxime (Table 3). Particularly trimethoprim-sulfamethoxazole forms fuzzy zone edges resulting in a broad variation of manual measurements (Tables 3, and 4). For trimethoprim-sulfamethoxazole the EUCAST reading guide for disk diffusion testing recommends to “ignore faint or haze growth up to the disk within a zone with otherwise clear zone edge” [21]. The definition of the zone edge and “faint or haze growth” is strongly dependent on factors like positioning of the plate, ambient light, or even the visual acuity of the investigator. Reading inhibition zones by a camera under standardised conditions and defining the zone edge by picture analysis with a well-defined software algorithm can help to standardise readings and enhance reproducibility and precision of AST reports. Other examples for reading difficulties are chromogenic compounds such as nitrofurantoin that appears as a yellow coloring of the agar hampering precise inhibition zone measurements. The size of the nitrofurantoin inhibition zone tends to be underestimated by the unaided eye and measurement variations are comparably high, frequently resulting in non-fulfilled quality control criteria (Table 4). Fully automated Sirscan readings solved these problems and resulted in low measurement variation along with zone diameters that were in agreement with EUCAST quality control criteria. Manual measurements of amikacin diameters in *S. aureus* ATCC 29213 and ertapenem diameters in *E. coli* ATCC 25922 tended to be higher than the quality control range. With fully automated Sirscan readings all measurements were in agreement with EUCAST quality control criteria. These examples illustrate the utility of fully automated zone diameter readings to enhance reproducibility and precision of the Kirby-Bauer method.

## Conclusions

Fully automated readings proved to be a useful tool to automate and standardise disk diffusion measurements improving the quality and reproducibility of AST reports. This is of particular interest in the light of decreasing and/or abandoning intermediate zones by EUCAST or CLSI and the associated need of more precise measurements to avoid interpretation errors.

## Competing interests

This work was supported by the University of Zurich. There are no competing interests to declare.

## Authors’ contributions

MH conceived of the study, performed the statistical analysis, and drafted the manuscript. RZ participated in data documentation and analysis. ECB, and participated in the study design and coordination and helped to draft the manuscript. All authors read and approved the final manuscript.
